# Bisleuconothine A, a bisindole alkaloid, inhibits colorectal cancer cell *in vitro* and *in vivo* targeting Wnt signaling

**DOI:** 10.18632/oncotarget.7190

**Published:** 2016-02-04

**Authors:** Ling-Mei Kong, Tao Feng, Yuan-Yuan Wang, Xing-Yao Li, Zhen-Nan Ye, Tao An, Chen Qing, Xiao-Dong Luo, Yan Li

**Affiliations:** ^1^ State Key Laboratory of Phytochemistry and Plant Resources in West China, Kunming Institute of Botany, Chinese Academy of Sciences, Kunming 650201, China; ^2^ University of the Chinese Academy of Sciences, Beijing 100049, China; ^3^ Harbin Institute of Technology (Weihai), Weihai 264209, China; ^4^ Present address: Georgia Regents University Health Sciences Campus, Augusta, Georgia 30912, USA; ^5^ Kunming Medical University, Kunming 650500, China

**Keywords:** Bisleuconothine A, inhibitor, Wnt signaling, colorectal cancer cells

## Abstract

Wnt signaling pathway is aberrantly activated in a variety of cancers, especially in colorectal cancer and small molecule antagonists of Wnt/β-catenin signaling are attractive candidates for developing effective therapeutics. In the present study, we identified Bisleuconothine A, a bisindole alkaloid with an eburnane-aspidosperma type skeleton, as a novel and selective Wnt signaling inhibitor by using a cell-based luciferase assay system. Our study found that Bisleuconothine A down-regulated the endogenous Wnt target gene expression through promoting phosphorylation of β-catenin and the subsequent inhibition of its nuclear translocation in HCT116 and SW480 colorectal cancer cells. *In vitro*, Bisleuconothine A inhibited cell proliferation through induction of apoptosis by increasing the cleavage of caspases in HCT116 and SW480 colorectal cancer cells. Moreover, *in vivo*, Bisleuconothine A dramatically suppressed tumor growth in HCT116 Xenograft. And further analysis showed that Bisleuconothine A suppressed the Wnt target gene expression in HCT116 Xenograft, which was associated with up-regulation of β-catenin phosphorylation and subsequent Wnt signaling inhibition. Taken together, our study indicated that bisindole alkaloids could be included as a new chemotype of small-molecule Wnt signaling inhibitors, and have great potential to be further developed for anti-tumor agents.

## INTRODUCTION

Wnt/β-catenin signaling is involved in a multitude of developmental processes and the maintenance of adult tissue homeostasis, as well as maintaining adult stem cells in a pluripotent state [1]. Wnts are a family of extracellular ligands that trigger a wide range of cellular responses upon receptor binding and activation [2]. In the absence of Wnt proteins, β-catenin is recruited into a destruction complex comprising adenomatous polyposis coli (APC), Axin, kinases casein kinase 1 (CKI) and glycogen synthase kinase-3β (GSK-3β). The destruction complex mediates sequential phosphorylation of β-catenin at S45 by CK1α and then at T41, S37 and S33 by GSK3 and targets β-catenin for the subsequent ubiquitination and proteasome degradation, and thereby maintaining low baseline cytosolic levels. Binding of Wnt proteins to Frizzled receptors inhibits GSK-3β and induces the disassembly of destruction complex and release of β-catenin, leading to accumulated cytoplasmic β-catenin and increased translocation of β-catenin into the nucleus, where it binds to the T-cell factor (TCF)/Lymphoid enhancer-binding factor (LEF) family of transcription factors [2, 3].

Mutations or deregulated expression of components of the Wnt pathway can induce diseases, most importantly cancer [4]. Over 94% of colorectal cancer cells have mutations in one or more members of the Wnt signaling pathway [5], of which 80% of sporadic colorectal cancers are associated with mutation of *APC* and 10% with mutation of *CTNNB1*, the gene encoding the protein of β-catenin [4]. Aberrant TCF/β-catenin signaling activity leads to the formation of cancer by altering expression of target genes that control cell proliferation, differentiation, migration and apoptosis [6], such as, Axin 2 [7], survivin [8], Cyclin D1 and c-Myc [9].

The broad involvement of Wnt signaling in disease has driven numerous studies to identify novel small molecules inhibitors that target different regulation levels of Wnt signaling. Despite of a couple of inhibitors reported with great potential, the clinical application of therapies to specifically target the Wnt pathway in cancer cells is still in infancy [1, 10].

Bisindole alkaloids have received considerable attention for their potential biological activity, including anti-tumor [11, 12], antimalarial [13], anti-inflammatory [14] and antibacterial [15, 16]. In our previous investigation, we isolated indole alkaloids from plants of genus *Melodinus* [12, 17–21], in which more than 200 monoterpenoid indoles with skeletons of aspidosperma, eburnane, condylocarpan, vincadine, scandine and bisindoles with two units of eburnane-aspidosperma, aspidosperma-scandine, aspidosperma-aspidosperma were reported with their cytotoxicity evaluation. However, few investigation on Wnt signaling pathway of bisindole was reported except cis-dihydroarcyriarubin C with only moderate inhibition on Wnt signaling transcription, suggesting that bisindole alkaloids might be novel candidates for Wnt signaling inhibitors [22]. In the present study, we identified bisindole alkaloids as Wnt signaling inhibitor by using a cell-based luciferase assay system, among which, Bisleuconothine A (BLA) showed the most potent activity. Bisleuconothine A, a bisindole with an eburnane-aspidosperma skeleton firstly reported from the bark of *Leuconotis griffithii*, exhibited cell growth inhibitory activity against various human cancer cell lines [23]. We showed that Bisleuconothine A, as a novel and selective Wnt signaling inhibitor, had great potential to be further developed as colorectal cancer therapeutics targeting the Wnt signaling.

## RESULTS

### Bisleuconothine A is a potent and selective inhibitor of the Wnt pathway

**Figure 1:**
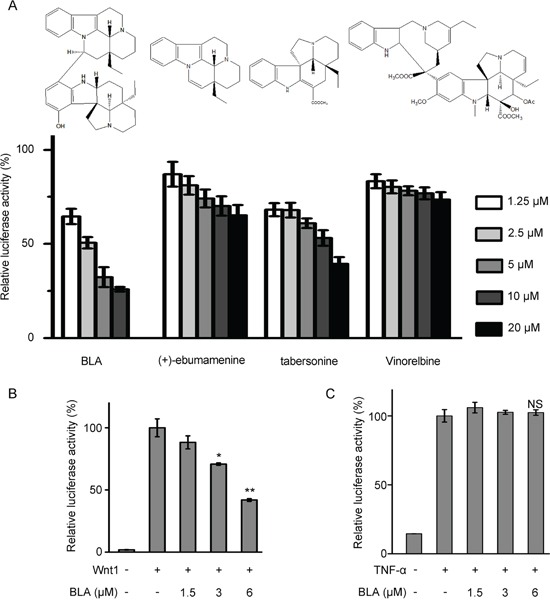
Bisleuconothine A is a potent and selective inhibitor of the canonical Wnt pathway **A.** The inhibitory activity of vinorelbine, Bisleuconothine A (BLA) and its derivatives on Wnt signaling. HEK293W cells in 96-well plates were incubated with indicated concentrations of different compounds for 24 h. The luciferase activity was then measured and normalized to the activity of the Renilla. The values represent the mean ± S.D. (n=3). **B.** HEK293T cells in 96-well plates were transiently transfected with Wnt1 (64 ng), SuperTop Flash construct (80 ng) and Renilla (8 ng) in each well. After incubation for 3 h, cells were treated with indicated concentrations of Bisleuconothine A for 24 h, and the luciferase activity was then measured and normalized to the activity of the Renilla. The values represent the mean ± S.D. (n=3). **C.** HEK293T cells in 96-well plates were transiently transfected with NF-κB Luc (150 ng) and Renilla (15 ng) in each well. After incubation for 3 h, cells were pretreated with indicated concentrations of Bisleuconothine A and stimulated with 25 ng/ml TNF-α for 24 h, and the luciferase activity was then measured and normalized to the activity of the Renilla. The values represent the mean ± S.D. (n=3). The significance was determined by Student's t test (*p<0.05, **p<0.01 vs. control).

To identify additional scaffolds of Wnt/β-catenin signaling inhibitors, a library consisting of about 4000 small molecules were screened by using a Wnt/β-catenin signaling reporter activity assay in HEK293W cells stably transfected with Wnt3a, Renilla and SuperTopflash luciferase (ST-Luc) [24]. The screening resulted in several primary hits, and one of which was Bisleuconothine A (Figure 1A). As shown in Figure 1A, incubation of HEK293W cells with Bisleuconothine A resulted in a dose-dependent decrease in Wnt3a induced ST-Luc transcription with an IC_50_ value of 2.55 ± 0.34 μM. To further confirm the inhibitory effect of Bisleuconothine A on the Wnt/β-catenin signaling, HEK293T cells were transiently transfected with the Wnt/β-catenin signaling reporter ST-Luc, Wnt1 and Renilla, and then treated with Bisleuconothine A for 24 h. As shown in Figure 1B, Wnt1 treatment resulted in an increase of the ST-Luc activity. Of note, Wnt1-induced ST-Luc activity was significantly decreased under Bisleuconothine A treatment with a calculated IC_50_ of 4.94 ± 0.10 μM (Figure 1B). To investigate the selectivity of Bisleuconothine A on Wnt signaling, the effect of Bisleuconothine A on NF-κB signaling was tested parallelly. HEK293T cells were transiently transfected with NF-κB-Luc (Beyotime Institute for Biotechnology) and Renilla. As shown in Figure 1C, TNF-α treatment significantly induced NF-κB transcriptional activity. However, Bisleuconothine A did not affect TNF-α-activated NF-κB reporter activity (Figure 1C), indicating that Bisleuconothine A preferentially targets Wnt signaling.

Bisleuconothine A is bisindole condensed by (+)-ebumamenine and a tabersonine derivative, and the cytotoxic activity against human cancer cell lines of their analogs have been investigated by our group previously [12, 17–21]. The results suggested that the cytotoxicity of hydroxyl substituent tabersonine decreased significantly, and the bioactive bisindole alkaloids invariably displayed greater potency compared to their monomeric alkaloids. To investigate the structure-activity relationship of Bisleuconothine A, we investigated the Wnt inhibition activities of the two constructive units of Bisleuconothine A, (+)-ebumamenine and tabersonine. However, neither of them exhibited Wnt signaling inhibitory activity in HEK293W cells, compared to Bisleuconothine A.

As a well-known anticancer chemotherapeutics, vinorelbine is also a bisindole alkaloid with similar structure with Bisleuconothine A. So we compared the Wnt signaling inhibitory activities of vinorelbine with Bisleuconothine A, and the dual-luciferase reporter assay in HEK293W cells showed that vinorelbine did not exhibit significant Wnt signaling inhibitory activity, indicating a novel anti-tumor mechanism of bisindole alkaloids.

### Bisleuconothine A attenuates Wnt signaling in colorectal cancer cell lines *in vitro*

**Figure 2:**
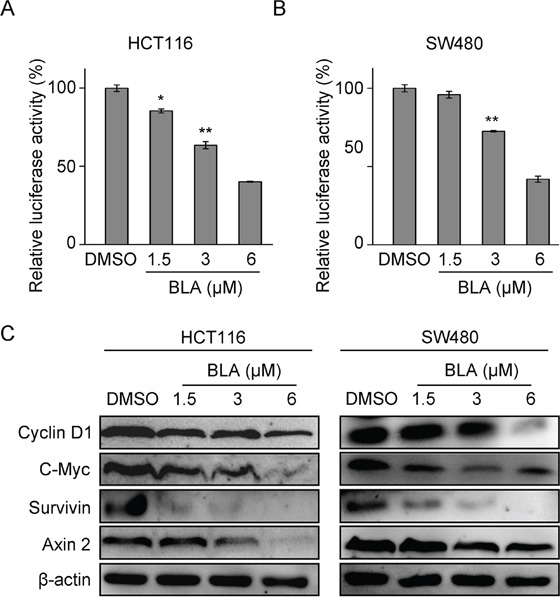
Bisleuconothine A attenuates the canonical Wnt pathway in colorectal cancer cells HCT116 **A.** and SW480 **B.** cells in 96-well plates were transiently transfected with SuperTop Flash construct (80 ng) and Renillaplasmids (8 ng) in each well. After incubation for 3 h, cells were treated with indicated concentrations of Bisleuconothine A (BLA) for 24 h, and the luciferase activity was then measured and normalized to the activity of the Renilla. The values represent the mean ± S.D. (n=3). **C.** Western blot analysis of Wnt signaling downstream target proteins in HCT116 and SW480 cells with β-actin used as the loading control.

To test whether Bisleuconothine A attenuates Wnt/β-catenin signaling in Wnt-dependent cancer cells, HCT116 and SW480 cells were transiently transfected with the Wnt/β-catenin signaling reporter ST-Luc and Renilla. The elevated ST-Luc activity in HCT116 and SW480 cells, due to Ser45 deletion mutation in β-catenin [25] and APC mutation [26], respectively, was significantly decreased by treatment with Bisleuconothine A (Figure 2A and 2B), and the IC_50_ value was 4.47 ± 0.16 μM and 4.94 ± 0.09 μM in HCT116 and SW480 cells respectively. We next investigated the effect of Bisleuconothine A on the expression of endogenous Wnt target genes Cyclin D1, c-myc, Axin 2 and survivin using Western blot analysis. As shown in Figure 2C, exposure of HCT116 and SW480 to increased concentrations of Bisleuconothine A for 24 h obviously suppressed the expression of cyclin D1, c-myc, Axin 2 and survivin.

### Bisleuconothine A promotes the phosphorylation and suppresses the nuclear translocation of β-catenin in colorectal cancer cells

**Figure 3:**
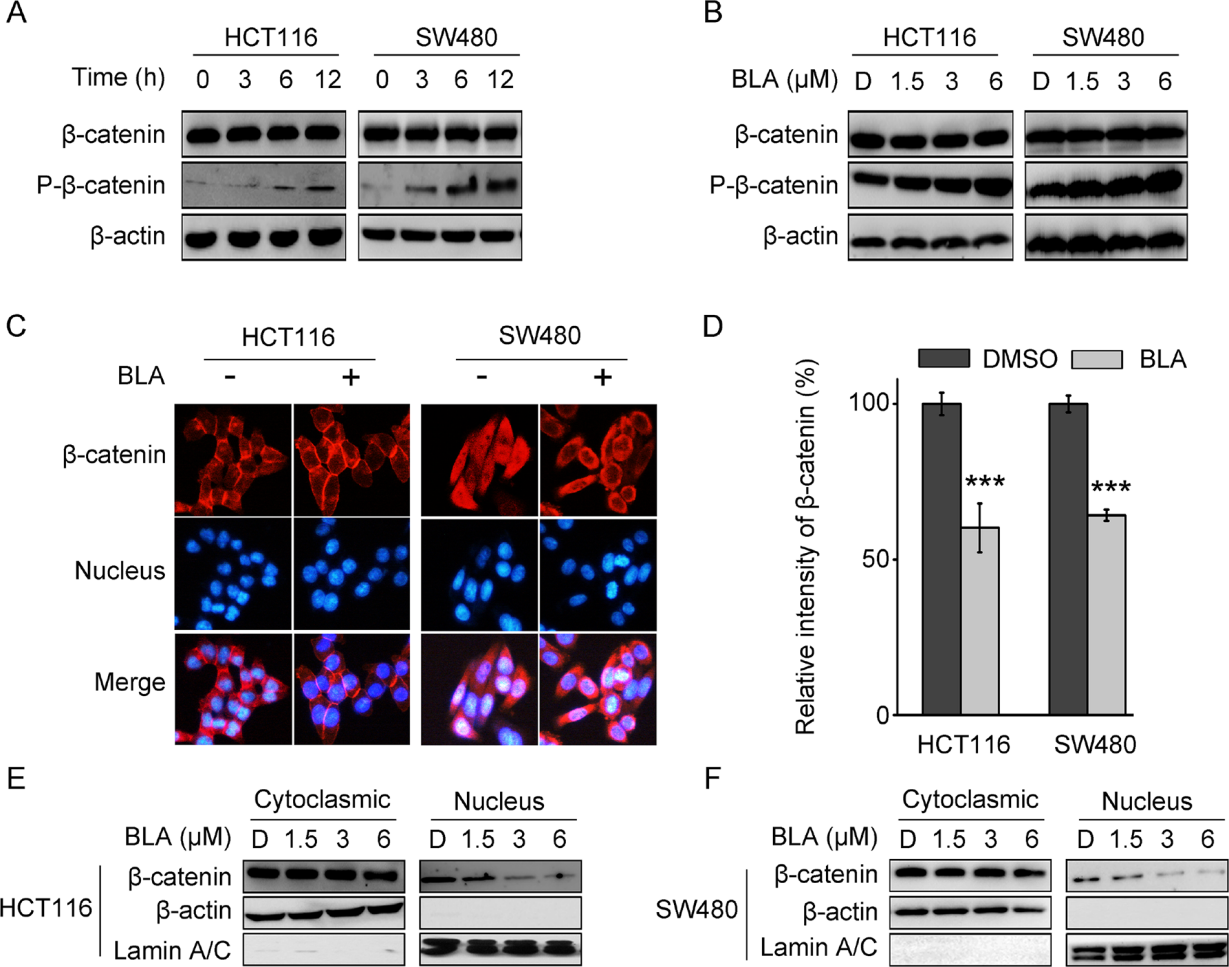
Bisleuconothine A promotes the phosphorylation and suppresses the nuclear translocation of β-catenin in colorectal cancer cells **A.** Western blot analysis of β-catenin and p-β-catenin (Ser33, Ser37 and Thr41) in HCT116 and SW480 cells treated with 3 μM of Bisleuconothine A (BLA) for 0 h, 3 h, 6 h and 12 h, respectively. β-actin was used as the loading control. **B.** Lysates from HCT116 and SW480 cells treated with DMSO (D), 1.5, 3 and 6 μM of Bisleuconothine A for 12 h were subjected to western blot analysis. The levels of β-catenin and p-β-catenin (Ser33, Ser37 and Thr41) were detected with β-actin used as the loading control. **C.** Representive figures of immunofluorescence staining. HCT116 and SW480 cells were incubated with or without Bisleuconothine A (3 μM). Distribution of β-catenin was examined by immunofluorescence staining. β-catenin and nucleus were recognized by the red and blue fluorescence, respectively. **D.** And the intensity of β-catenin in the nucleus of cells treated with DMSO and Bisleuconothine A was analyzed and quantified. All the values represent the mean ± S.D. (n=3). The significance was determined by Student's *t* test (***p<0.001 vs. control). HCT116 **E.** and SW480 **F.** cells were incubated with DMSO (D), 1.5, 3 and 6 μM of Bisleuconothine A for 12 h. The levels of β-catenin in cytoplasmic fraction and nucleic fraction of HCT116 and SW480 cell lysates were analyzed by Western Blot. β-actin and Lamin A/C is the loading control of cytoplasmic fraction and the nucleic fraction, respectively.

In the Wnt/β-catenin signaling pathway, β-catenin response transcription is largely dependent on the level of β-catenin, which is regulated by the destruction complex [27]. Thus, the protein levels of total β-catenin and phosphorylated β-catenin were investigated. To test whether treatment of Bisleuconothine A induces β-catenin phosphorylation, lysates from cells incubated with Bisleuconothine A with incremental time of Bisleuconothine A were subjected to Western blot analysis. As shown in Figure 3A and 3B, exposure of HCT116 and SW480 cells to Bisleuconothine A induced the Ser33/37/41 phosphorylation of β-catenin in a time and dose dependent manner.

Induction of phosphorylation of β-catenin led to the inhibition of the nuclear translocation of β-catenin, thus, the distribution of β-catenin was analyzed by immunofluorescence assays. As shown in Figure 3C and 3D, Bisleuconothine A significantly inhibited the endogenous nuclear translocation of β-catenin in HCT116 and SW480 cells, with dominant reduction in the intensity of β-catenin in the nucleus of cells treated with Bisleuconothine A. Consistently in the Western Blot assay (Figure 3E and 3F), the level of β-catenin in the cytoplasmic fraction of HCT116 and SW480 cells hardly changed, whereas the distribution of β-catenin in the nucleus was attenuated treated with Bisleuconothine A.

### Bisleuconothine A attenuates the canonical Wnt pathway in colorectal cancer cells at the level of the destruction complex

**Figure 4:**
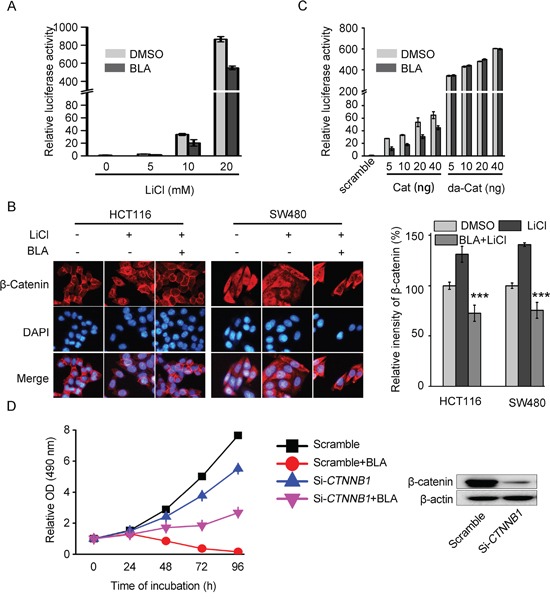
Bisleuconothine A attenuates the canonical Wnt pathway in colorectal cancer cells at the level of or upstream the destruction complex **A.** HEK293T cells in 96-well plates were transiently transfected with SuperTop Flash construct (80 ng) and Renilla plasmids (8 ng) in each well. After incubation for 3 h, cells were treated with 6 μM of Bisleuconothine A (BLA) for 1 h and stimulated with indicated concentrations of LiCl for 24 h, and the luciferase activity was then measured and normalized to the activity of the Renilla. The values represent the mean ± S.D. (n=3). **B.** Representive figures of immunofluorescence staining. HCT116 and SW480 cells were incubated with or without Bisleuconothine A (3 μM) for 6 h in the absence or presence of LiCl for 6 h. Distribution of β-catenin was examined by immunofluorescence staining. β-catenin and nucleus were recognized by the red and blue fluorescence, respectively. The intensity of β-catenin in the nucleus of cells treated with DMSO, LiCl and Bisleuconothine A in the presence of LiCl was analyzed and quantified. All the values represent the mean ± S.D. (n=3). The significance was determined by Student's *t* test (***p<0.001 vs. LiCl). **C.** HEK293T cells in 96-well plates were transiently transfected with SuperTop Flash construct (80 ng) and Renilla plasmids (8 ng) and different concentration of Cat (Flag-β-catenin) or da-Cat (S37A Flag-β-catenin) in each well. After incubation for 3 h, cells were treated with 6 μM Bisleuconothine A for 24 h, and the luciferase activity was then measured and normalized to the activity of the Renilla. The values represent the mean ± S.D. (n=3). **D.** The SW480 cells were transfected with *CTNNB1* small interfering RNA for 36 h, and the relative OD (490 nm) of every point normalized to that of 0 h respctively was documented at 0, 24, 48, 72 and 96 h in the absence or presence of Bisleuconothine A (BLA) (2.5 μM), respectively. The values represent the mean ± S.D. (n=3).

LiCl, an inhibitor of GSK-3β, could down-regulate the level of phosphorylation of β-catenin and subsequently induce nuclear translocation of β-catenin [28]. As shown in Figure 4A, LiCl significantly increased the ST-Luc in HEK293T cells, while pretreatment of HEK293T cells with Bisleuconothine A inhibited LiCl-induced activation of Wnt signaling. These results indicated that Bisleuconothine A exerted their effects at the level of the β-catenin destruction complex (GSK-3β/AXIN/APC) or upstream of it. As an inhibitor of GSK-3β, LiCl promoted the nuclear translocation of β-catenin (Figure 4B). Of note, pretreatment of HCT116 and SW480 cells with 3 μM Bisleuconothine A for 6 h dramatically inhibited LiCl-induced nuclear translocation of β-catenin. HEK293 cells were transiently transfected with ST-Luc/Renilla and with plasmids encoding scramble, full-length β-catenin or β-catenin with S37A (da-Cat). Due to the mutation of S37, targeting β-catenin for phosphorylation, ubiquitination and degradation, the β-catenin (S37A) directly translocated into the nucleus, thereby constantly activating the Wnt signaling pathway without being phosphorylated and subsequent degradation [29, 30]. As shown in Figure 4C, the activity of ST-Luc with transfection of full-length β-catenin was reduced by incubation with Bisleuconothine A. However, β-catenin with S37A-induced Wnt activation was not attenuated by Bisleuconothine A. Thus, Bisleuconothine A inhibits the Wnt signaling pathway through induction of phophorylation of β-catenin and subsequent nucleus translocation inhibition of β-catenin. Besides, siRNA mediated knock-down of β-catenin reduced the sensitivity of SW480 cells to Bisleuconothine A remarkably, deduced from the cell growth curves (the red curve and the pink curve in Figure 4D), further confirming that β-catenin is required for the anti-tumor activity of Bisleuconothine A.

### Bisleuconothine A inhibited cell proliferation through induction of apoptosis by increasing the cleavage of caspases in colorectal cancer cells

**Figure 5:**
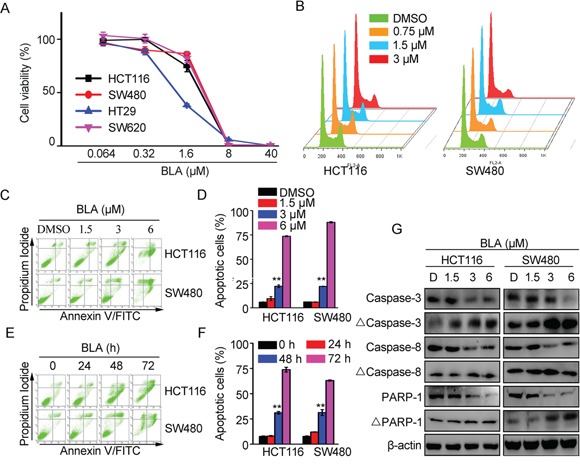
Bisleuconothine A inhibited cell proliferation through induction of apoptosis by increasing the cleavage of caspases in colorectal cancer cells **A.** HCT116, SW480, HT29 and SW620 cells in 96-well plates were treated with Bisleuconothine A (BLA) at different concentrations or with DMSO as a control for 48 h. Cell viability was measured by MTS assay and cell proliferation was normalized against the control cells. **B.** HCT116 and SW480 cells incubated with indicated concentrations of Bisleuconothine A were subjected to Cell Cycle analysis. **C.** HCT116 and SW480 cells were incubated with Bisleuconothine A at concentrations of 1.5, 3 and 6 μM for 48 h. Apoptosis was analyzed by Annexin V-FITC/PI staining. **D.** All the values represent the mean ± S.D. (n=3). The significance was determined by Student's *t* test (*p<0.05, **p<0.01 vs. control). **E.** HCT116 and SW480 cells were treated with Bisleuconothine A at 3 μM for 24, 48 and 72 h. Apoptosis were analyzed by Annexin V-FITC/PI staining. **F.** All the values represent the mean ± S.D. (n=3). The significance was determined by Student's t test (*p<0.05, **p<0.01 vs. control). **G.** Lysates from HCT116 and SW480 cells treated with DMSO (D), 1.5, 3 and 6 μM of Bisleuconothine A for 24 h were subjected to western blot analysis. The level of caspase-3, cleaved caspase 3 (Δcaspase 3), caspase 8, Δcaspase 8, PARP-1 and ΔPARP-1 were detected with β-actin used as the loading control.

Given that Wnt signaling is closely associated with cell proliferation and Bisleuconothine A can suppress the Wnt signaling in colorectal cancer cells, we then explored the effect of Bisleuconothine A on cancer cell proliferation with MTS assay. As shown in Figure 5A, Bisleuconothine A inhibited the proliferation of HCT116, SW480, HT29 and SW620 colorectal cancer cells, and the IC_50_ values were 2.74 ± 0.22, 3.18 ± 0.08, 1.09 ± 0.06 and 3.05 ± 0.17 μM, which were comparable to those shown to attenuate the activity of Wnt signaling.

Cyclin D1 is an important regulator of the cell cycle that is overexpressed in colorectal cancers as a consequence of activated TCF/β-catenin signaling [31]. Our study found that Bisleuconothine A inhibited Wnt signaling and its target gene Cyclin D1 protein expression of in HCT116 and SW480 cells (Figure 2C). To confirm whether the anti-proliferative effect of Bisleuconothine A was due to induction of cell cycle arrest, propidium iodide (PI) staining and flow cytometry analysis of cells were performed. As shown in Figure 5B, Bisleuconothine A induced weak G0/G1 cell cycle arrest. Besides, Bisleuconothine A-induced apoptosis was investigated, and the results showed that Bisleuconothine A exhibited a markedly concentration- and time-dependent apoptosis inducing effect on HCT116 and SW480 cells (Figure 5C and 5D). Additionally, extension of the bisleuconothine A treatment time increased the percentage of apoptotic cells (Figure 5E and 5F). With western blotting assay, our results demonstrated that dose-dependent proteolytic cleavages of caspase 3, caspase 8, and PARP-1 were detected in Bisleuconothine A treated HCT116 and SW480 cells (Figure 5G).

### Bisleuconothine A sulfate suppressed tumor growth through downregulation of Wnt signaling in Xenograft mouse model *in vivo*

Subcutaneous Xenograft model was used to investigate the anti-tumor capacity of Bisleuconothine A *in vivo*. To increase the water solubility of Bisleuconothine A, the sulfate of Bisleuconothine A (Bisleuconothine A-S, BLA-S) (Supplementary Figure 1A) was prepared and the inhibitory effects of Bisleuconothine A sulfate on Wnt signaling in HEK293W cells and growth of several colorectal cancer cells were evaluated (Supplementary Figure 1B, 1C). As shown in Supplementary Figure 1, the growth and Wnt inhibitory activity of Bisleuconothine A sulfate was consistent with that of Bisleuconothine A.

**Figure 6:**
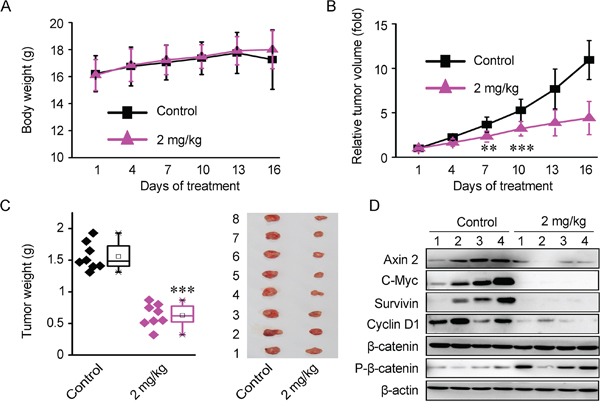
Bisleuconothine A sulfate suppresses colorectal cancer cells Xenografts growth through downregulation of Wnt signaling in HCT116 Xenografts mouse model *in vivo* **A.** Each mouse was injected with 5×10^6^ of HCT116 cells. One week later, tumor bearing mice were treated intraperitoneally with normal saline as control or 2 mg/kg Bisleuconothine A sulfate daily for 16 days. The body weight of each mouse was monitored for 16 days. Each point represented a mean ± S.D. (n=8). **B.** Effect of Bisleuconothine A sulfate on relative tumor volume of HCT116 Xenograft tumors. Each point represented a mean ± S.D. (n=8). **C.** The external appearance of tumors is shown and tumor weight was measured at the 16th day. Each point represented a mean ± S.D. (n=8). The significance was determined by Student's *t* test (**p<0.01 vs. control). **D.** Effect of Bisleuconothine A sulfate on expression of Wnt target genes, as well as β-catenin and p-β-catenin (Ser33, Ser37 and Thr41) in tumor tissues were determined by Western blot analysis. β-actin was used as loading control.

In the *in vivo* test, 7 days after implantation, the BALB/c mice carrying established HCT116 Xenograft were randomly assigned into control and experimental group. The mice were treated daily with 2 mg/kg of Bisleuconothine A sulfate by intraperitoneal injection and injection of normal saline as controls. As shown in Figure 6A, the experimental group treated with 2 mg/kg of Bisleuconothine A sulfate did not show toxicity to the mice as no difference in weight was observed between control and Bisleuconothine A sulfate-treated mice. While, relative tumor volume and tumor weight were significantly decreased with treatment with Bisleuconothine A sulfate (Figure 6B and 6C). Importantly, the relative tumor volume was significantly decreased started from 7 days treatment with 2 mg/kg/d of Bisleuconothine A sulfate (*p*<0.05) compared with controls (Figure 6B). At the end of the experiment, the tumors in control mice grew to approximately 1750 mm^3^, whereas the tumors of mice treated with 2 mg/kg Bisleuconothine A sulfate were approximately 590 mm^3^. Meanwhile, tumor weight (Figure 6C) was reduced by 59% in mice receiving 2 mg/kg/d of Bisleuconothine A sulfate compared with controls. These results demonstrate that Bisleuconothine A sulfate has potent anti-tumor activity in HCT116 colorectal Xenograft.

Of note, the expression of Wnt target genes Axin 2, c-Myc, Cyclin D1 and survivin was significantly reduced in the tumor tissues treated with Bisleuconothine A sulfate, as well as the induction of Ser33/37/41 phosphorylation of β-catenin (Figure 6D). Collectively, our results indicate that Bisleuconothine A inhibits tumor growth through attenuating Wnt signaling.

## DISCUSSION

Aberrant activation of the Wnt/β-catenin signaling pathway is involved in the development and progression of various cancers, especially colorectal *cancers* [4, 9]. Wnt/β-catenin signaling has become a potential target in cancer treatment. In colorectal cancers where Wnt/β-catenin signaling is frequently activated by mutated *APC* or β-catenin, it seems that, for antitumor efficacy, the ideal antagonist of the pathway would be targeting the transcriptional complex of TCF and β-catenin. However, there are recently some studies showing that, at least in some cases, targeting the upstream components of the Wnt signaling pathway can also play a role. As the key component of Wnt signaling, the stabilization of β-catenin is a potential target, which is regulated by the destruction complex. Recently, several small molecules targeting the destruction complex have been discovered. XAV939 [32], IWR [33], JW55 [34], J67 and J74 [35] increase Axin levels through inhibiting the activity of tankyrase, while the compound Pyrvinium [36] enhances casein kinase to promote the phosphorylation of β-catenin and disturbs the stabilization of β-catenin.

Bisleuconothine A, a bisindole alkaloid with an eburnane-aspidosperma type skeleton, exhibited cell growth inhibitory activity against various human cancer cell lines, but little is known about its mechanism of action [23]. In the present study, by using a reporter gene system [24], our screening resulted in the identification of Bisleuconothine A as a novel inhibitor of β-catenin/TCF transcription activity. The inhibitory effect of Bisleuconothine A in LiCl-induced cells and in *APC* mutant cells indicated that the compound might act on the destruction complex or upstream this level. The molecular target of Bisleuconothine A and the detailed mechanisms needs further investigation.

Bisindole alkaloids, well known as inhibitors of tubulin polymerization, are extensive in clinical application, such as Vinblastine and Vincristine, as well as their derivatives Vindesine and Vinorelbine [37]. Despite of the similarity in structure, the dual-luciferase assay showed that Vinorelbine did not exhibit Wnt signaling inhibitory activity, suggesting Bisleuconothine A inhibits the Wnt signaling specifically and the mechanism studies of anti-tumor and Wnt signaling inhibitory activity of Bisleuconothine A enriched the anti-cancer mechanisms of bisindole alkaloids.

In conclusion, we identified Bisleuconothine A as a novel and selective antagonist of Wnt signaling for the first time and disclosed the mechanism of the anti-proliferative effect of Bisleuconothine A on colorectal cancer cells *in vitro* and *in vivo*. Bisleuconothine A suppressed Wnt/β-catenin signaling by inhibition of nuclear translocation of β-catenin via a mechanism dependent of β-catenin phosphorylation and independent of *APC*. Taken together, our study suggested that Bisleuconothine A might be developed into therapeutic agents against various cancers bearing aberrant upregulation of Wnt signaling.

## MATERIALS AND METHODS

### Cell culture

The human colon cancer cell lines (HCT116, SW480, HT29 and SW620) and HEK293T were purchased from the Shanghai Institute of Biochemistry and Cell Biology, Chinese Academy of Sciences (Shanghai, China). The cells were grown in medium (DMEM for HCT116, SW480, SW620 and HEK293T cells, RPMI 1640 medium for HT29), supplemented with 10% fetal bovine serum (FBS), 100 units/ml penicillin G sodium and 100 μg/ml streptomycin (HyClone). HEK293W cells [24] (Wnt3a and luciferases stably transfected) were cultured in DMEM medium, supplemented with 10% fetal bovine serum (FBS), 100 units/ml penicillin 100 μg/ml streptomycin (HyClone), 100 μg/ml G418 (Sigma-Aldrich) and 100 μg/ml Hygromycin B (Sigma-Aldrich) as previously reported. All the cells were incubated at 37°C, 5% CO_2_ in a humidified atmosphere.

### Cell transfection and luciferase reporter assay

Cells were seeded in 96-well plates. After overnight culture, for one well, HEK293T cells transiently transfected with 80 ng Wnt/β-catenin pathway responsive firefly luciferase reporter plasmid SuperTOPFlash (ST-Luc), 8 ng of Renilla reporter plasmid (Promega) and 64 ng mouse Wnt1. In HCT116 and SW480 cells, the cells were transiently transfected with ST-Luc and Renilla. After 3 h incubation, cells were treated with various concentrations of Bisleuconothine A for 24 h and then the cells were lysed. Both luciferase and Renilla activities were measured using the Dual-Luciferase Reporter Assay kit (Promega, Madison, WI). The luciferase activity was normalized to the Renilla activity.

### Western blotting assay

Cells were harvested and lysed in SDS sample buffer (62.5 mM Tris-HCl, pH6.8, 10% glycerol, 2% SDS, 50 mM DTT and 0.01% bromphenol blue). Lysates were subjected to SDS-PAGE and transferred to PVDF membranes (Millipore). Membranes were blocked with 5% nonfat milk in Tris-buffered saline/0.1% Tween-20 and incubated at 4°C overnight with the following antibodies: anti-Axin 2, anti-Cyclin D1, anti-c-Myc, anti-Survivin, anti-caspase 3, anti-cleaved caspase 3, anti-caspase 8, anti-PARP-1 (Santa Cruz, CA), anti-Lamin A/C (Epitomics), anti-β-catenin (BD Biosciences), anti-phopho-β-catenin (Cell signaling Technology), and against β-actin (Santa Cruz, CA), followed by the corresponding horseradish peroxidase-conjugated secondary antibodies. Proteins of interest were visualized and imaged under chemi-luminescent detection using LASmini 4000 (GE Healthcare).

### Immunofluorescence staining

Cells were grown for 12 h before treatment. Drug-treated cells were fixed in 4% paraformaldehyde for 20 min. After being washed with PBS, the slides were treated by 0.1% Triton-X and blocked with 3% BSA in PBS. Then the slides were incubated with anti-β-catenin antibody (BD Biosciences) overnight, washed with PBS, and incubated with corresponding FITC conjugated secondary antibody (Sigma-Aldrich) for 1 h. The cells were then incubated with DAPI (4′, 6-diamidino-2-phenylindole) for 10 min and observed under microscopy (Eclipse Ti, Nikon).

### Nuclear and cytoplasmic fractionation

Collected cells were re-suspended in lysis buffer A (10 mM HEPES, 10 mM KCl, 1.5 mM MgCl_2_, 0.5 mM DTT, pH 7.9, EDTA-free protease inhibitor cocktail (Roche, Indianapolis, IN, USA)) and incubated on ice for 10 min. After microcentrifuged, the cells were lysed in buffer A with 0.2% Nonidet P-40 and for 6 minutes on ice. After being microcentrifuged for 5 min at 500 g, the supernatants were collected as cytoplasmic extracts. Pellets were washed with lysis buffer A without NP-40 and resuspended in lysis buffer B (20 mM HEPES, pH 7.9, 420 mM NaCl, 0.5 mM DTT, 0.2 mM EDTA, and 25% glycerol), and incubated for 30 min on ice. After being centrifuged at 12,000 g for 10 min, supernatants were collected.

### Small interfering RNAs

Duplex siRNAs with two thymidine residues (dTdT) at the 3′-end of sequence were synthesized at GenePharma (Shanghai, China). The target sequences were as follows [38]:

*CTNNB1*: 5′-AGCUGAUAUUGAUGGACAG-3′.

Scrambled control: 5′-UUCUCCGAACGUGUCACGU-3′.

SiRNAs were transfected into SW480 cells using Lipofectamine 2000.

### MTS assay

Cytotoxicity of compounds was determined by MTS method. Briefly, 5×10^3^ cells were plated in 96-well plates 12 h before treatment and continuously exposed to test compounds for 48 h. Then MTS (Promega) was added to each well. The samples were incubated at 37°C for 1∼4 h and the optical density (OD) was measured at 490 nm using a microplate reader (Bio-Rad Laboratories). The IC_50_ values are calculated from appropriate dose-response curves.

### Cell cycle analysis

HCT116 and SW480 cells (2×10^5^ cells) were incubated with test compounds for 24 h respectively. Ccells were collected and washed twice with PBS. Cells were fixed with 70% ethanol overnight. Fixed cells were washed with PBS, and then stained with 50 μg/ml propidium iodide (PI, Sigma-Aldrich) solution containing 50 μg/ml RNase A (Sigma-Aldrich) for 30 min in dark at room temperature. Fluorescence intensity was analyzed by FACSCalibur flow cytometer (BD Biosciences, Franklin Lakes, NJ). The percentages of the distributions in distinct phases of cell cycle were determined using FlowJo V 7.6.1 software.

### Cell apoptosis analysis

Cell apoptosis was analyzed using the Annexin V-FITC/PI Apoptosis kit (BD Biosciences, Franklin Lakes, NJ) according to the manufacturer's protocols. Cells were seeded in 6-well plates at a density of 2×10^5^ cells/well. After indicated treatments, the cells were collected and washed twice with cold PBS, and then resuspended in a binding buffer containing Annexin V-FITC and propidium iodine (PI). After incubation for 15 min at room temperature in the dark, the fluorescent intensity was measured using a FACSCalibur flow cytometer (BD Biosciences, Franklin Lakes, NJ).

### Xenograft studies

All animal experiments were carried out under an Institutional Animal Care and Use Committee-approved protocol and institutional guidelines for the proper and humaneuse of animals. Three-week-old female BALB/c-nude Mice were purchased from Vital River Laboratory Animal Technology Co. Ltd, and were kept in a pathogen-free environment. For the HCT116 xenograft, 5×10^6^ HCT116 cells were injected subcutaneously to the right flank of the BALB/c-nude mice. When the tumors reached a volume of ∼130 mm^3^, 7 days after implantation, the mice were randomly assigned into two groups (eight mice in each group) and treatments were given daily as follows: (a) intraperitoneal injection of 2 mg/kg Bisleuconothine A sulfate, or (b) intraperitoneal injection of normal saline. Tumors were measured in three dimensions using calipers, and tumor volume (in mm^3^) was calculated using the following formula: tumor volume (mm^3^) = (L× W^2^)/2, where L= length in mm, and W= width in mm. Body weight was recorded regularly during the experiment. After treatments, the mice were sacrificed and the tumors were isolated. Then the tumors were weighed and frozen at −80°C for subsequent western blotting assay.

## SUPPLEMENTARY FIGURE


