# Supplementary material for: Bisleuconothine A, a bisindole alkaloid, inhibits colorectal cancer cell in vitro and in vivo targeting Wnt signaling
Source: Oncotarget. 2016 Feb 4;7(9):10203–14. doi: 10.18632/oncotarget.7190 (PMC4891114; doi:10.18632/oncotarget.7190)
Supplement: Supplementary file 1 [file oncotarget-07-10203-s001.pdf]

SUPPLEMENTARY FIGURE

The synthesis, *in vitro* Wnt signaling inhibitory activity and cytotoxicity of Bisleuconothine A sulfate.

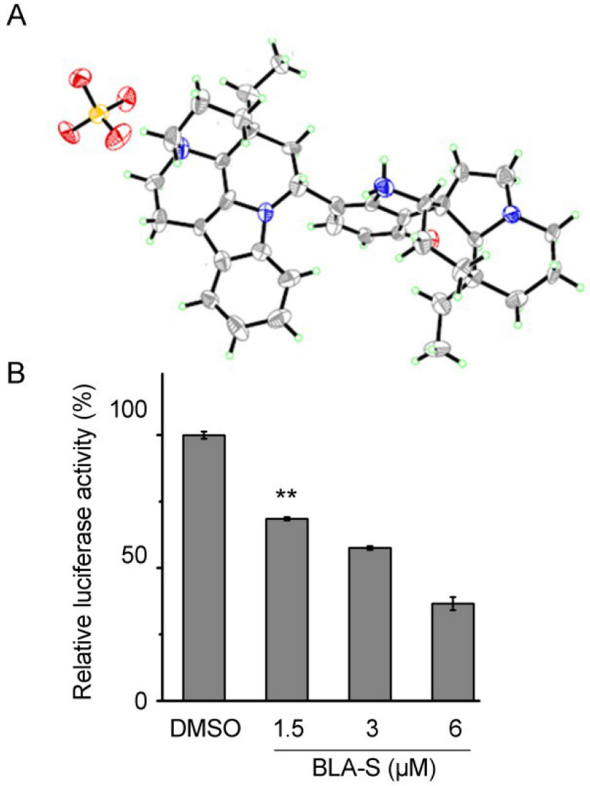

Compounds	IC_{50} (Mean ± SD, μM)
BLA-S	3.86±0.14
BLA	2.55±0.34

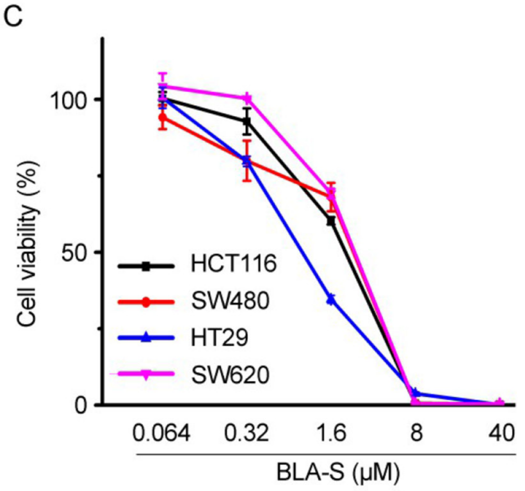

Cell lines	IC_{50} (Mean±SD, μM)	
	BLA-S	BLA
HCT116	2.47±0.35	2.74±0.22
SW480	2.46±0.20	3.18±0.08
HT29	0.93±0.04	1.09±0.06
SW620	2.51±0.07	3.05±0.17

Supplementary Figure S1: The *in vitro* Wnt inhibitory activity and cytotoxicity of Bisleuconothine A sulfate. A. X- ray structure of Bisleuconothine A sulfate (BLA-S). **B.** HEK293W cells in 96-well plates were incubated with 1.5, 3 and 6 μM Bisleuconothine A sulfate for 24 h, respectively. The luciferase activity was then measured and normalized to the activity of the Renilla. The IC_{50} values were calculated and the values represent the mean ± S.D. (n=3). **C.** HCT116, SW480, HT29 and SW620 cells in 96-well plates were treated with Bisleuconothine A sulfate at different concentrations or with DMSO as a control for 48 h. Cell viability was measured by MTS assay and cell proliferation was normalized against the control cells. The IC_{50} values were calculated and the values represent the mean ± S.D. (n=3).
